# Using the endoscopic snare to facilitate two-port laparoscopic appendectomy

**DOI:** 10.1007/s00464-025-11743-z

**Published:** 2025-05-06

**Authors:** Mohamed Farid, Azza Baz, Mohamed Riad, Ashraf Abdelmonem Elsayed, Ahmed Salah Arafa, Rasha S. Elsayed, Alaaedin Ramadan, Ibrahim A. Heggy, Mostafa M. Elaidy

**Affiliations:** 1 General Surgery Department, Zagazig University, Zagazig City, Egypt; 2 General Surgery Department, Al-Ahrar Teaching Hospital, Zagazig City, Egypt

**Keywords:** Two ports, Laparoscopic appendectomy, Novel approach, Endoscopic snare

## Abstract

**Background:**

Laparoscopic appendectomy is now the gold-standard treatment for acute appendicitis, requiring three ports for a classic procedure. Recent laparoscopy improvements aim to minimize surgical trauma and improve cosmetic quality through smaller, fewer portal incisions, such as two-port laparoscopic appendectomy, resulting in reduced postoperative pain. We aimed in this study to describe a novel technique to facilitate two-port laparoscopic appendectomy using the endoscopic snare.

**Patients and methods:**

The data for a total of 85 patients, who underwent the two-port laparoscopic appendectomy using the endoscopic snare, at two research centers in Zagazig city, Egypt, from July 2022 till July 2023, is retrospectively analyzed. Overall length of hospital stay was the primary outcome, and the duration of operation and patient cosmetic satisfaction were secondary endpoints.

**Results:**

All the 85 laparoscopic procedures were completed without difficulty. The mean operative time was 43.78 ± 8.46 min (minimum: 34 min, maximum: 57 min). Length of hospitalization was 1.12 ± 0.74 days (min: 1 day, max: 2 days). No major complications were encountered. Four cases of minor postoperative complication occurred, in which the patient developed port site infection, which was completely resolved at one week postoperatively.

**Conclusion:**

Laparoscopic appendectomy, using only two ports and endoscopic snare, is generally feasible and has been linked to high patient satisfaction and excellent cosmetic outcomes.

For both simple and complex cases of acute appendicitis, laparoscopic appendectomy is now considered the gold-standard treatment, and in order to perform a classic laparoscopic appendectomy, three ports are needed [1].

Even with the recent improvements in laparoscopy, there is still interest in minimizing surgical trauma and improving cosmetic quality through smaller and fewer portal incisions [2].

The less invasive two-port laparoscopic appendectomy treatment leads to a shorter incision, reduced postoperative pain, and improved cosmetic outcome [3].

Our goal aims to evaluate the effectiveness and safety of performing the two-port laparoscopic appendectomy using the endoscopic snare as a novel approach.

## Patients and methods

### Study design

This is a two research center retrospective study that included all patients with inclusion criteria and the two-port laparoscopic appendectomy using the endoscopic snare was used during the period from July 2022 to July 2023 (the study period) at two research centers in Zagazig city, Egypt.

### Study approval

Ethical approval for this study was granted by the Institutional Review Board of the Zagazig University, with an IRB registration number is ZU-IRB#759/8–12–2024.

### The inclusion criteria

All patients for whom the two-port laparoscopic appendectomy using the endoscopic snare was used during the period from July 2022 to July 2023 (the study period).

### Exclusion criteria

1. Patients with acute perforated appendicitis.
2. Patient with appendicular mass or phlegmon.
3. Patients with appendicular abscess.

### Workup and selection of patients

Data were collected from the archived medical records of the surgical departments of 2 research centers and were retrospectively analyzed for all patients with inclusion criteria and the two-port laparoscopic appendectomy using the endoscopic snare was used during the period from July 2022 to July 2023 (the study period).

**Fig. 1:**
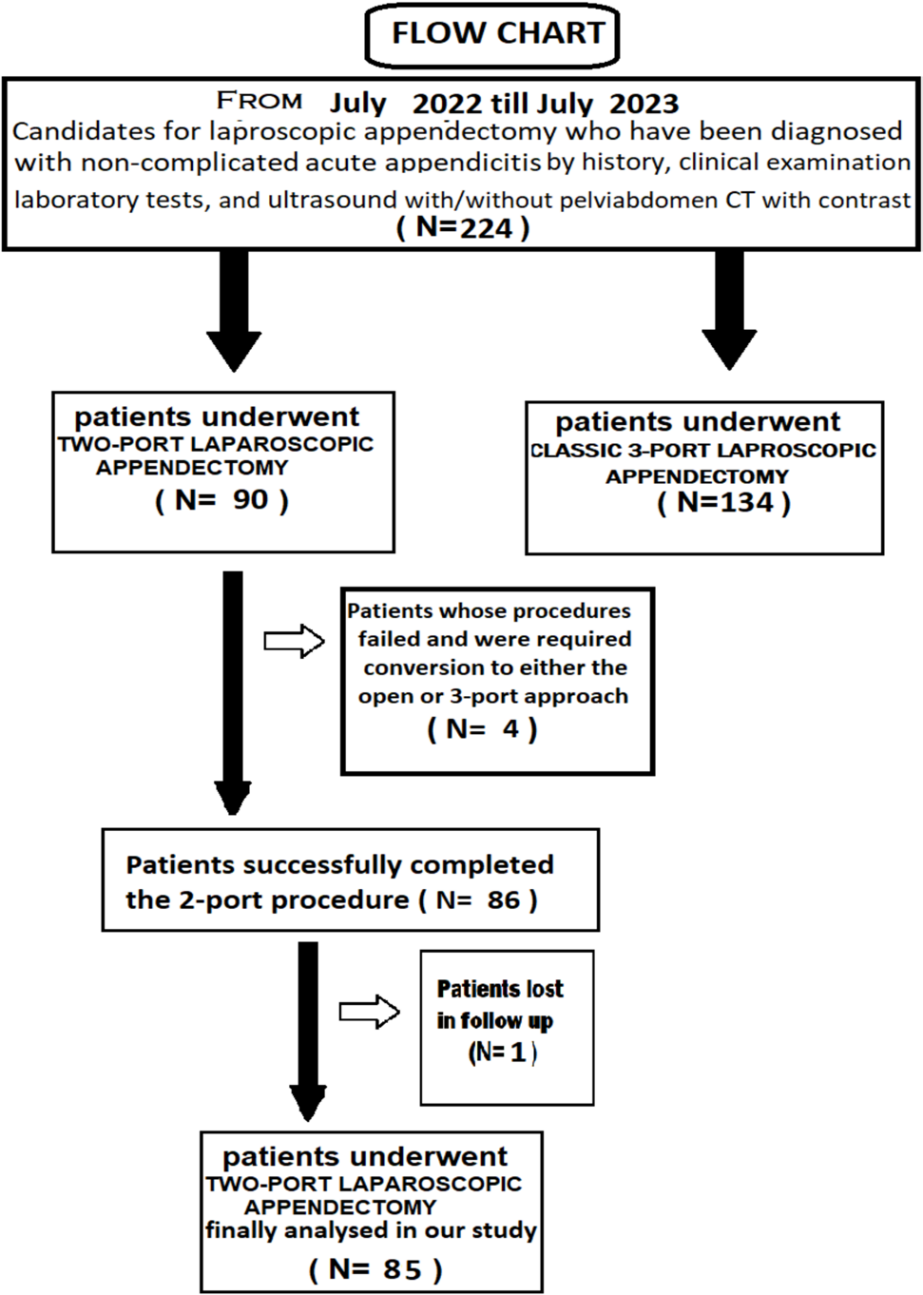
The flowchart for the allocation process throughout the study

A total number of 224 patients with the diagnosis of acute non-complicated appendicitis and performed laparoscopic appendectomy were analyzed in this study period [151 patients at the emergency Surgery Unit in Zagazig University Hospitals and 73 patients at the emergency Surgery Unit at Al-Ahrar teaching hospital]. 90 patients were operated with the two-port laparoscopic appendectomy, and 134 patients were operated with the standard 3 ports. Only 4 patients of those operated with the two-port laparoscopic appendectomy are failed and required conversion to either open or standard 3-port laparoscopy. During the follow-up period, only one patient was lost in his visits, so, the final number of patient analyzed in our study for the two-port laparoscopic appendectomy was 85 patients. See the flowchart for the allocation process throughout the study (Fig. 1).

Clinical diagnosis of non-complicated acute appendicitis was obtained based on the patient's full history (including a characteristic recent non-neglected right lower abdomen or periumbilical discomfort, nausea/vomiting, anorexia, or a low grade fever,…) and physical examination (right iliac fossa tenderness or guarding with a positive McBurney's sign, cough test,…). In every patient, laboratory tests such as complete blood count (CBC), C-reactive protein (CRP), pregnancy test for female patient in childbearing period were requested and interpreted. Also, an ultrasound with or without pelvis-abdomen CT scan with contrast was performed in all patients to support the diagnosis and to exclude other possible causes of acute abdomen, and the most important was to exclude patients with complicated appendicitis, including appendicular abscess, phlegmon, or generalized peritonitis.

Prior to surgery, all patients signed informed written consent forms. The patients' characteristics and demographic data, the operative time, hospital stay, peri-operative complications or morbidity, time to return to work, and postoperative outcomes were all noted. We assessed the degree of patients’ satisfaction of residual scarring using a 5-point Likert scale (1 = very unsatisfied, 2 = not satisfied, 3 = acceptable, 4 = satisfied, 5 = very satisfied). This satisfaction score were obtained by a separate individual by telephoning every patient during their follow-up period. The wound satisfaction score (WSS) was recorded for each patient after three weeks and three months postoperatively to assess the patient’s satisfaction with his/her scar [4].

### The surgical procedure

A routine preoperative anesthetic evaluation was used to prepare the patients. General anesthesia was used for all procedures. A surgeon on the left side of the patient and an assistant on the right side of the surgeon carried out the operations. Depending on the situation and the surgeon's preference, a transverse skin incision was made at the umbilicus and a Veress needle or the optical port was used to create the pneumoperitoneum with a pressure of about 14–15 mm Hg of CO2 and a 10-mm optical camera with a 0° angle was placed through the umbilical trocar. Although a 5-mm optical camera could be used, but we preferred to use the 10 mm one to facilitate the surgical specimen extraction retrogradely under vision through the 10-mm port while withdrawing the telescope. The 5-mm trocar was then introduced through the suprapubic region (n = 58) or the right iliac fossa (n = 37), depending on the situation and the surgeon's preference to facilitate intra-operative manipulation and without any recorded difference in postoperative wound satisfaction score. Patients were placed in the Trendelenburg position, while the table was tilted to the left by about 15–20 degrees.

**Fig. 2:**
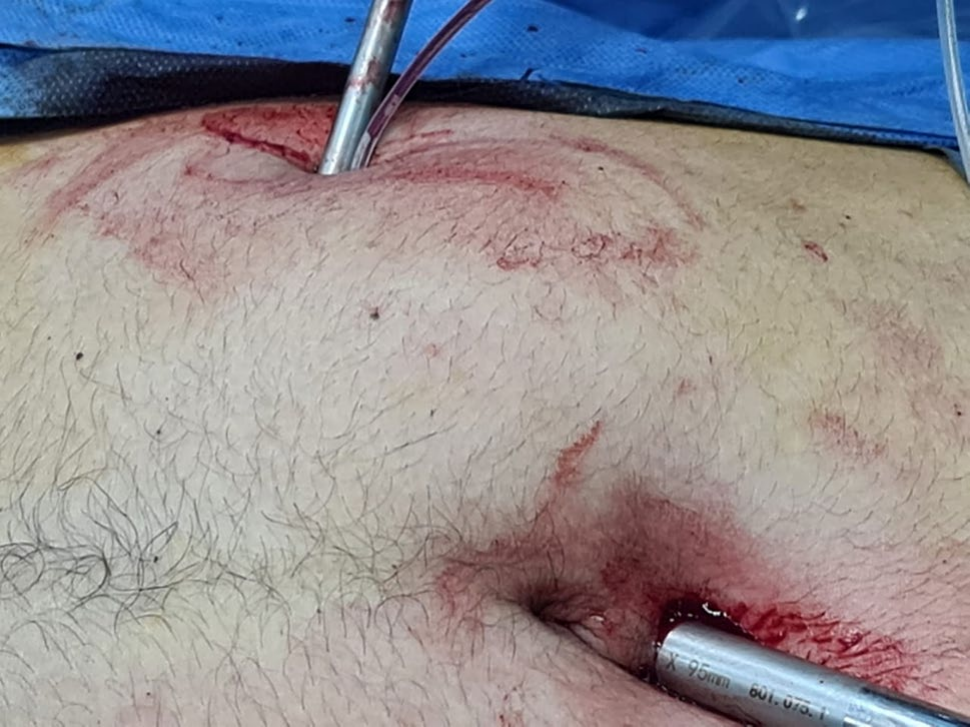
Alongside the 5-mm suprapubic trocar, the same skin incision was used to introduce the endoscopic snare

**Fig. 3:**
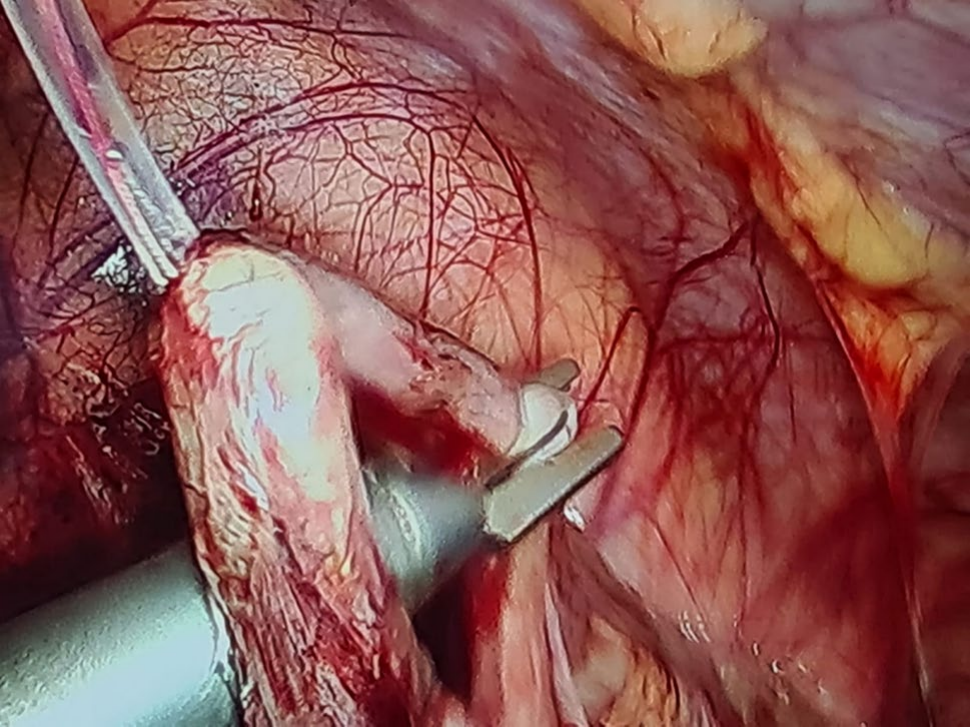
With the aid of the endoscopic snare the appendix was readily and successfully manipulated, just like an endo-grasper

**Fig. 4:**
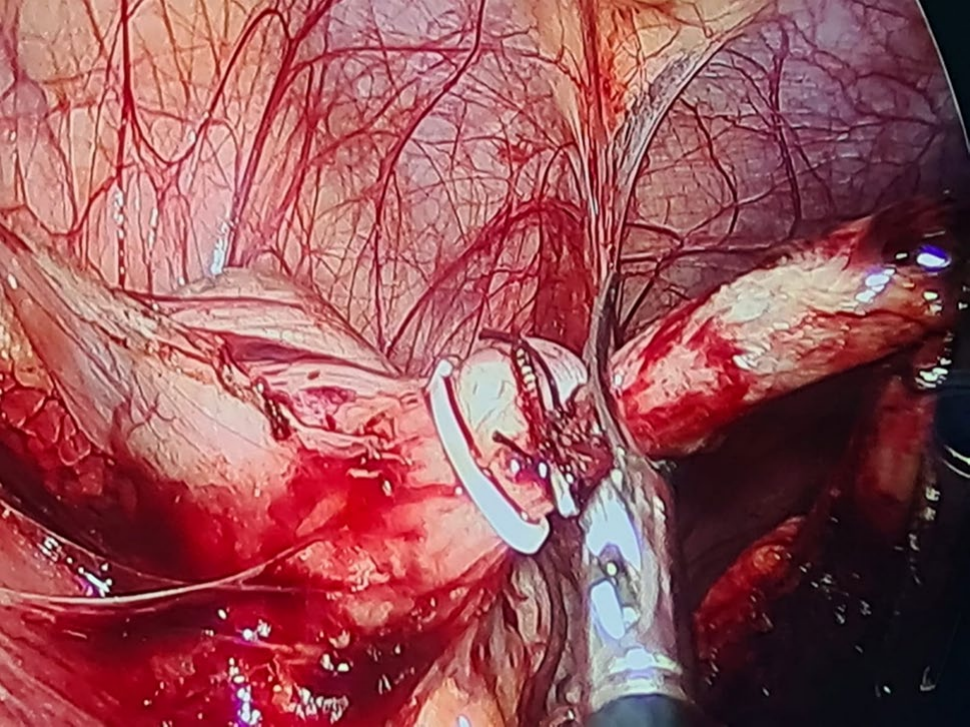
A 5-mm endoscopic titanium or polymer endo-clips were used to ligate the appendix near its base

A thorough diagnostic laparoscopy was started with routinely in all cases to confirm the diagnosis of acute appendicitis and to exclude other possible cause of the illness. Alongside the 5-mm trocar, the same skin incision was used to introduce the endoscopic snare (Fig. 2). With the aid of the endoscopic snare, the appendix was readily and successfully manipulated, just like an endo-grasper (Fig. 3). A LigaSure or a Harmonic vessel-sealing device was used to cut and safely separate the meso-appendix, according to the availability and the surgeon preference. A 5-mm endoscopic titanium or polymer endo-clips, according to the situation and availability, were used to ligate the appendix near its base by applying 2 staples on the base and one staple on the stump (Fig. 4), and appendectomy was completed. The surgical specimen was extruded retrogradely under vision through the 10-mm port while withdrawing the telescope. The fascia was closed with 2/0 vicryl and the skin was closed with 4/0 sub-cutaneous vicryl sutures. All specimens were sent postoperatively for histopathological examination.

### Statistical analysis

The categorical variables were presented as a number (%), while the continuous variables were presented as the mean ± SD & median (range). Using the Shapiro–Wilk test, continuous variables were examined for normality. It was deemed statistically significant when the P value was less than 0.05. SPSS version 22.0 for Windows (IBM Corp., Armonk, NY, USA) was used for all statistics.

## Results

We retrospectively investigated the hospital records of a total of 85 (48 female and 37 male) patients at two research centers in Zagazig city; 53 patients at the emergency Surgery Unit in Zagazig University Hospitals and 32 patients at the emergency Surgery Unit at Al-Ahrar teaching hospital from July 2022 till July 2023 that underwent two-port laparoscopic appendectomy using the endoscopic snare. Patients with acute perforated appendicitis or with appendicular mass, phlegmon, or with appendicular abscess are excluded. The specimens’ histopathological reports showed the diagnosis of acute catarrhal appendicitis (n = 71) and acute suppurative appendicitis (n = 14).

Patients’ demographic characteristics are collected and analyzed (Table 1). The mean age was 27.12 ± 9.24 years (minimum age = 17 and maximum age = 56), and the mean BMI was 28.37 ± 6.51 kg/m2 (minimum BMI = 18.25 and maximum BMI = 45.35).

**Table 1:** Patients' characteristics

Patients' characteristics	(N = 85)	
	No	%
Sex		
Male	37	43.53%
Female	48	56.47%
Age (years)		
Mean ± SD	27.12 ± 9.24 years	
Median (Range)	28 (17 – 56)	
BMI		
Mean ± SD	28.37 ± 6.51 kg/m2	
Median (Range)	(18.25–45.35)	

Categorical variables were expressed as number (percentage); continuous variables were expressed as mean ± SD & median (range)

**Fig. 5:**
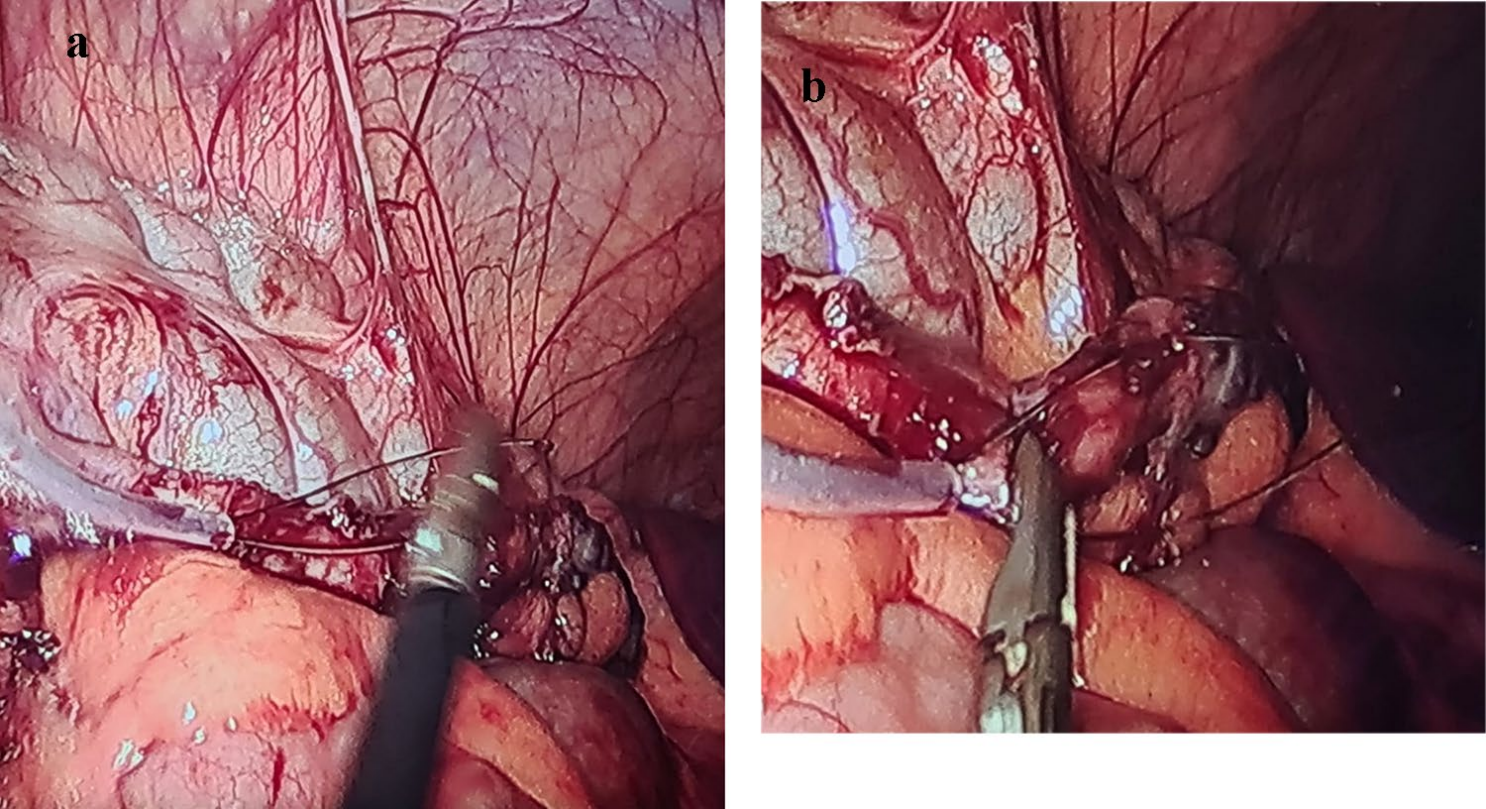
**a** and **b** The 5-mm port instrument facilitated this process of snare appendicular suspension by guiding and encircling the appendix

Most of our patients underwent successful surgery and for only 4 patients (4.44%), the two-port technique was failed and required conversion either to open surgery nor the traditional three-port technique were required. Those 4 patients had relative difficulties with two-port technique and either were necessitated further dissection of the meso-appendix or anatomically deep seated adherent retro-cecal appendicitis. The mean operative time was 43.78 ± 8.46 min (minimum: 34 min, maximum: 57 min). It was interestedly noticed that the mean operative time in patients operated with the standard 3-port technique was 41.56 ± 7.38 min (minimum: 32 min, maximum: 54 min), which was nonstatistically significant lower than the two-port laparoscopic appendectomy. This relatively nonstatistically significant longer operative time while using the endoscopic snare is related to the fact that the snare lakes rigidity and its manipulation takes a very few minutes early intraoperatively till ligating and suspending the appendix, then no surgical ergonomic issues to be faced. The 5-mm port instrument facilitated this process of snare appendicular suspension by guiding and encircling the appendix (Fig. 5a and b). It was noticed also that by elapsing time, the learning curve of operating surgeon was increasing, and this longer time was improving. Only 3 patients who had a postoperative infection at the periumbilical port site region are recovered with medical treatment and dressing and all are related to cutting the appendicular stump without overlying staple. The mean hospitalization period was 1.12 ± 0.74 days (min: 1 day, max: 2 days).

The wound satisfaction score Mean ± SD was 4.73 ± 0.32, which was confirmed by patients after three weeks and was 4.86 ± 0.28 after 3-month postoperatively to measure the cosmetic results for our port site wounds (Table 2).

**Table 2:** Patients’ operative data and outcome

	(*N* = 85)	
	No	%
Operation duration (min.)		
Mean ± SD	43.78 ± 8.46 min	
(Range)	(minimum: 34 min—maximum: 57 min)	
Severe adhesions		
Absent	204	93.6%
Present	14	6.4%
Hospital stay (days)		
Mean ± SD	1.12 ± 0.74 days	
(Range)	(minimum: 1 day—maximum: 2 days)	
	After 3 weeks	After 3 months
Wound satisfaction score		
Mean ± SD	4.73 ± 0.32	4.86 ± 0.28

Categorical variables were expressed as number (percentage); continuous variables were expressed as mean ± SD & median (range)

## Discussion

For the traditional three-port laparoscopic appendectomy, there are a number of literatures that outline how to minimize this minimally invasive surgical technique as reducing the quantity of ports, reducing the sizes of the ports, or describing a modified approach to suspended the appendix [5].

There are numerous established methods for doing this two-port laparoscopy appendectomy. Roberts described the "puppeteer technique," which involves the surgeon's left hand suspending a suture that emerges from the right iliac fossa. Thirteen out of fourteen instances in this puppeteer technique study had effective surgeries, with patients reporting improved cosmetic outcomes and less postoperative pain [6].

Another method was employed for a 36 patients (with failure in 5 cases) by Yeung et al., who inserted an intravenous cannula through the abdominal wall, leaving the plastic cannula in place as the future knot pusher, after the needle was removed. Using a catheter, one end of a ligature is inserted into the peritoneum. Through one of the ports, the intraperitoneal end is gripped and extracted. A suture loop is created by creating a sliding knot extra-corporeally. To bring the loop back into the peritoneum, gently tug on the opposite end. There was no scarring at the catheter site, they said the McBurney point is perfect for reducing infections and problems, and it was particularly helpful in instances with inflamed appendices [7].

In a different modification by Ateş et al., for the trans-abdominal sling suture technique for 38 patients (with failure in 3 cases), where he used a single port but contained a two channels (one for the laparoscope), the appendix is lifted after being gripped and separated from the surrounding tissues using a single grasper or dissector. The appendix was dragged against the abdominal wall after a suture was percutaneously (with a needle) inserted from the right lower quadrant into the peritoneal cavity and then passed through the mesoappendix [8].

Donmez et al. employed a needle grasper as a percutaneous organ holding device at the McBurney point for a total of 32 patients, allowing him to manipulate the appendix's position with his left hand. The appendix was skeletonized when the LigaSure device cauterized the mesoappendix through the suprapubic trocar. An endoloop was used to suture the appendix at the radix through the suprapubic trocar and an appendectomy was carried out [9].

Bonatti utilized two 5-mm ports and a needle grasper for a total of 39 patients, in an attempt to avoid inserting the classic 10/12-mm trocar. A 5-mm port should be used to begin this approach. After exploring the abdominal cavity, the surgeon can determine the following steps, such as additional port placements, based on anatomical findings and the appendix's appearance. He frequently fully skeletonizes the appendix so that it can be removed via a smaller incision [1].

The mean operative time in our study was 43.78 ± 8.46 min (minimum: 34 min, maximum: 57 min) and in patients operated with the standard 3-port technique was 41.56 ± 7.38 min (minimum: 32 min, maximum: 54 min), which was nonstatistically significant shorter than the two-port laparoscopic appendectomy. This was relatively near results from Augustin G and Morshed G, where the operative time ranged from 37 to 54 min (mean = 50 min) and from 45 to 60 min, respectively [5, 10].

Meanwhile, results from Olijnyk et al. and Donmez et al. showed relatively longer operative time where the mean operation time was 64.5 min and 57.03 ± 3.814 min (minimum: 48 min, maximum: 68 min), respectively [2, 9].

The failure and conversion rate in our study was only 4 patients (4.44%), which is less than results from Yeung et al. with failure in 5 cases of their total 36 patients (13.88%) and from Ateş et al. with failure in 3 cases of their total 38 patients (7.89%) [7, 8].

In our study, only 3 patients (3.52%) who had a postoperative infection at the port site region, and this was similar to results from Donmez et al. where they reported postoperative infection at the port site region in only one patient (2.94%) from a total of 34 patients in their study, and less than results from Panait et al. where they mentioned a postoperative infection and periumbilical cellulitis in only one patient (12.25%) from a total of 8 patients in their study [9, 11].

To the best of our knowledge, we present in our study the first literature to utilize the endoscopic snare as an organ holding device for the appendix to facilitate the two-port laparoscopic appendectomy. Also, because our study was performed in two research centers simultaneously, we have the highest number of operated patients (n = 85) using the two-port laparoscopic appendectomy approach in comparison to other mentioned before published literatures.

We believe that the patient will find this modified intracorporeal two-port approach to be a highly satisfactory cosmetic operation that is both safe and feasible. Although this is not a state-of-the-art surgery, some patients with physically "easier" forms of appendicitis and/or earlier presentations may benefit from it in facilities equipped with traditional laparoscopic equipment.

## Conclusion

Laparoscopic appendectomy, using only two ports and endoscopic snare, is feasible and has been linked to high patient satisfaction and excellent cosmetic outcomes. We think that using fewer and smaller incisions in an effort to minimize surgical trauma is beneficial to patients as evidenced by the wound satisfaction score, which was confirmed by patients after three weeks and after 3 months postoperatively to measure the cosmetic results for our port site wounds. The results of this study from two research institutions should encourage other surgeons to attempt this approach, especially for a common and reasonably easy operation like laparoscopic appendectomy. In our research, we found that the endoscopic snare can be useful for many minimally invasive laparoscopic procedures in addition to appendectomy.

## Data Availability

All data generated or analyzed during this study are included in this article. Further enquiries can be directed to the corresponding author.
